# Neural substrates of shared visual experiences: a hyperscanning fMRI study

**DOI:** 10.1093/scan/nsab082

**Published:** 2021-06-28

**Authors:** Ayumi Yoshioka, Hiroki C Tanabe, Motofumi Sumiya, Eri Nakagawa, Shuntaro Okazaki, Takahiko Koike, Norihiro Sadato

**Affiliations:** Department of Cognitive and Psychological Sciences, Graduate School of Informatics, Nagoya University, Nagoya 464-8601, Japan; Japan Society for the Promotion of Science, Tokyo 102-0083, Japan; Department of Cognitive and Psychological Sciences, Graduate School of Informatics, Nagoya University, Nagoya 464-8601, Japan; Japan Society for the Promotion of Science, Tokyo 102-0083, Japan; Division of Cerebral Integration, Department of System Neuroscience, National Institute for Physiological Sciences (NIPS), Okazaki 444-8585, Japan; Division of Cerebral Integration, Department of System Neuroscience, National Institute for Physiological Sciences (NIPS), Okazaki 444-8585, Japan; Division of Cerebral Integration, Department of System Neuroscience, National Institute for Physiological Sciences (NIPS), Okazaki 444-8585, Japan; Division of Cerebral Integration, Department of System Neuroscience, National Institute for Physiological Sciences (NIPS), Okazaki 444-8585, Japan; Division of Cerebral Integration, Department of System Neuroscience, National Institute for Physiological Sciences (NIPS), Okazaki 444-8585, Japan

**Keywords:** joint attention, mentalizing, intentionality, mirroring system, hyperscanning fMRI

## Abstract

Sharing experience is a fundamental human social cognition. Since visual experience is a mental state directed toward the world, we hypothesized that sharing visual experience is mediated by joint attention (JA) for sharing directedness and mentalizing for mental state inferences. We conducted a hyperscanning functional magnetic resonance imaging with 44 healthy adult volunteers to test this hypothesis. We employed spoken-language-cued spatial and feature-based JA tasks. The initiator attracts the partner’s attention by a verbal command to a spatial location or an object feature to which the responder directs their attention. Pair-specific inter-individual neural synchronization of task-specific activities was found in the right anterior insular cortex (AIC)–inferior frontal gyrus (IFG) complex, the core node of JA and salience network, and the right posterior superior temporal sulcus, which represents the shared categories of the target. The right AIC-IFG also showed inter-individual synchronization of the residual time-series data, along with the right temporoparietal junction and dorsomedial prefrontal cortex—the core components for mentalization and the default mode network (DMN). This background synchronization represents sharing the belief of sharing the situation. Thus, shared visual experiences are represented by coherent coordination between the DMN and salience network linked through the right AIC-IFG.

## Introduction

Sharing experience with each other is a fundamental human ability that enables culture (Tomasello *et al.*, 2005). Seeing, which is a form of perception, consists of two components: the visual experience and the objects that result in the visual experience (Searle, 1983). Perceptual experience is defined as the mental state directed at or of objects and states of affairs in the world, known as intentionality^1^ (Searle, 1983). Visual experience consists of directness and representative contents caused by the object, which is formulated as follows: ‘Agent-Attitude-Proposition’ (Baron-Cohen, 1995). For example, by directing attention toward the color of the flower, the visual experience can be written as follows: ‘I see the flower is yellow’, where ‘I’ is the agent, ‘see’ is the attitude (that is, the epistemic mental state toward the feature) and ‘the flower is yellow’ is the proposition (that is, the representative contents describing the semantic knowledge of the feature). The proposition ‘the flower is yellow’ is the primary representation defined as the direct semantic relation with the world. By including the mental state expression term, the sentence does not represent the world, but indicates representation. Thus, this formulation is called the M-representation (meta-representation) (Baron-Cohen, 1995). Therefore, sharing a visual experience involves sharing the M-representation caused by the same object.

Sharing a visual experience starts with attending to the same object, known as joint attention (JA). JA is the ability to coordinate attention between interactive social partners on a third significant object through eye contact, pointing, showing and other behavior, including language (Mundy, 2018). By sharing spatial attention toward an object as a reference point, JA enables one to share referential relations with a partner, leading to an alignment of their cognitive engagement in the situation, that is, perspective-taking (Liszkowski, 2018). According to Searle (1983), visual experience from different perspectives can be shared through the belief that ‘I am seeing it as part of our seeing it’ (pp. 70). Thus, the belief that they are seeing the same thing is shared through inferences of the belief of others, that is, mentalizing.

JA is theorized to be a precursor to mentalizing in the developmental trajectory driven by linguistic interaction (Mundy, 2018). Language provides JA with the system for framing attention, allowing for ideational JA, which forms and maintains shared experiences (Bruner, 1995). Verbally mediated JA is different from gaze-cued JA in that the shared attentional focus is not limited in the spatial location. Through gaze-cued JA, partners share their attentional focus, which is spatially fixed by the visual cue of the partner’s gaze. On the other hand, verbally mediated JA enables participants to share the object’s feature such as color, number and shape. These attentional foci are invisible, more abstract than the spatial location of the object. Thus, more abstract inference of the mental status of the partner (directedness of the attention; Are you attending to the color?) is required.

Previously, we found that the sharing experience during a JA task through eye-gaze was represented by neural synchronization of the task-specific activity (Koike *et al.*, 2019) as well as of the residual time series (Saito *et al.*, 2010; Tanabe *et al.*, 2012) in the right anterior insular region (AIC)–inferior frontal gyrus (IFG) region. Recent theoretical and experimental approaches suggest that inter-individual synchronization represents the forward model or prediction (Friston and Frith, 2015; Miyata *et al.*, 2021). According to the predictive coding theory, neuronal representations in higher cortical hierarchies predict the representations in lower levels (Mumford, 1992; Rao and Ballard, 1999; Friston, 2008). The comparison of top-down predictions (forward model) with representations at the lower level forms a prediction error fed back up the hierarchy to update higher representations. This recursive exchange of signals suppresses prediction error at every level to provide a hierarchical explanation for sensory inputs (Friston and Frith, 2015). According to the predictive coding account, both self action optimization and action inference of others require forward model or top-down prediction (Kilner *et al.*, 2007): The same forward model used to predict the sensorial effects of one’s own actions can also be used as a constraint for decoding the actions of others (Friston, 2005; Kilner *et al.*, 2007). Considering that the spontaneous neural activity reflects the internal model of the environment (Berkes *et al.*, 2011), the residual time-series synchronization may represent the forward model. As the comparison of top-down forward model with the lower representation generates the prediction error, the task-specific neural synchronization likely represents the prediction error.

Given the mentalizing network, included in the default mode network (DMN), represents the higher-order model for inference of self and other’s mental states (Andrews-Hanna, 2012), we hypothesized that shared visual experience is represented by inter-individual neural synchronization of the neural representation of mentalizing with JA-related substrates as its subsystem. To test this hypothesis, we conducted a hyperscanning functional magnetic resonance imaging (fMRI) study, utilizing spoken-language-cued spatial and feature-based JA tasks without gaze exchange.

## Materials and methods

### Participants

A total of 44 healthy adult volunteers (20 men, 24 women; 22 pairs, age = 21.27 ± 2.38, mean ± standard deviation years) participated in this study. Before the experiment, we assigned same-sex participants that had never seen each other beforehand to pairs. All participants were right-handed, according to the Edinburgh Handedness Inventory (Oldfield, 1971). None of the participants had a history of neurological or psychiatric illness. The protocol was approved by the ethical committee of the National Institute for Physiological Sciences (Okazaki, Japan). The participants gave their written informed consent before the experiment.

### Experimental procedure

#### Hyperscanning MRI system.

**Fig. 1.**
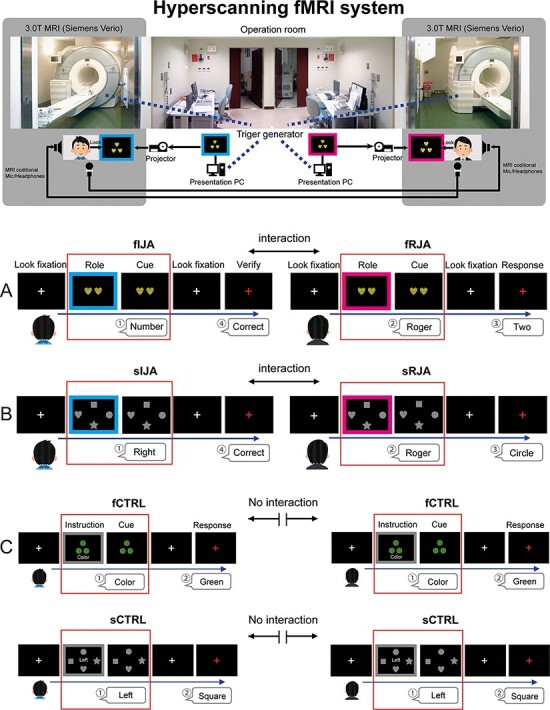
Experimental setup. (A) The feature-based JA condition. In the Role phase, the participant that is looking at the screen with a cyan frame is the initiator, and the participant with a magenta frame is the responder. The objects have four dimensions such as number (1, 2, 3 or 4), shape (star, heart, circle or square), color (red, blue, yellow or green) and pattern (stripe, border, check or dot). The initiator freely chooses one of the four dimensions (2.5 s). After the frame disappears (the Cue phase), IJA informs the chosen aspect by utterance and the responder says ‘Roger’ in the same period (2.5 s). In the Response and Verify phase, the responder replies with the characteristic of this dimension, and the initiator judges and feeds back, verbally, ‘correct’ or ‘incorrect’ (2.5 s). (B) The sJA condition: The gray four target objects (star, heart, circle or square) are displayed on above, below, left and right from the center of the screen. (C) The feature-based and spatial attention control (solo) condition (fCTRL or sCTRL): The frame color was gray, and participants perform this task without reference to their partners. Brain activity in the Role phase and Cue phase (surrounded by a red frame) was analyzed.

To measure the neural activation between pairs of participants, we used two MRI scanners equipped with a standard 32-channel phased array coil (Magnetom Verio 3T, Siemens, Erlangen, Germany; Figure 1). The two MRI scanners were combined with online video cameras (custom made by NAC Image Technology, Yokohama, Japan, and Panasonic System Solutions Japan Co. Ltd., Tokyo, Japan), microphones and headphones (Opto ACTIVE II, Kobatel, Yokohama, Japan). This setup allowed a reciprocal live interaction of pairs with utterance.

#### Stimulus presentation.

The visual stimuli for the JA tasks were generated using Presentation software (Neurobehavioral Systems, Berkeley, CA, USA). Video images of participant’s faces were captured using an online video camera system and combined using a Picture-in-Picture system (NAC Image technology and Panasonic System Solutions Japan Co. Ltd., Tokyo, Japan). The combined visual stimuli were projected using a liquid crystal display projector (CP-SX 12000J, Hitachi Ltd., Tokyo, Japan) onto a half-transparent screen that was placed on the scanner bed approximately 190.8 cm from the participants’ eyes. They were presented at a visual angle of 13.06° × 10.45° (Koike *et al.*, 2016). The video images of participant’s faces were used only in the self-introduction before the experiment and were not presented in the JA task. Participants were able to communicate with each other using their voices in real time.

#### MRI data acquisition.

MRI time-series data were acquired using ascending-order T2*-weighted, gradient-echo echo-planar imaging (EPI) with the multiband sequence developed at the University of Minnesota, MN, USA (Feinberg *et al.*, 2010; Moeller *et al.*, 2010; Xu *et al.*, 2013). Each volume consisted of 36 slices, each 3.0-mm thick with a 0.5-mm gap, to cover the entire cerebral cortex and the cerebellum. Images were taken at the first 500 ms (acquisition time, TA) of the 2,500 ms repetition time (TR), and the next 2 s was silent (i.e. no scanner noise). During the silent period, participants were prompted to talk to each other to avoid speech-related motion artifact. The flip angle (FA) generated was 80°, and the echo time (TE) was 30 ms. The multiband acceleration factor was 5. The field of view (FOV) was 192 mm, in-plane matrix size was 64 × 64 pixels, and size of one voxel was 3 mm × 3 mm × 3 mm. For the JA experiments, we acquired 175 volumes (approximately 7 min) per run. For anatomical reference, T1-weighted high-resolution images were obtained with three-dimensional (3D) magnetization-prepared rapid-acquisition gradient echo (MP-RAGE) sequences (TR = 1,800 ms, TI = 900 ms, TE = 1.98 ms, FA = 9°, 208 slices, thickness = 1 mm, FOV = 256 mm, voxel dimensions = 1 mm × 1 mm × 1 mm).

#### JA tasks.

In this experiment, all the tasks were mediated verbally. In the JA tasks, the participants were assigned to one of the two roles (Figure 1). One was the initiator of joint attention (IJA) that spontaneously attracted the partner’s attention to a specific place (i.e. spatial JA; sJA) or a feature of the object (i.e. feature JA; fJA). The other was the responder of joint attention (RJA) that received the verbal signal uttered by the partner and directed their attention to the same place or feature of an object as the partner. The roles of the initiator and responder were specified using colors during the stimulus presentation period. The cyan frame of the screen was used to indicate the initiator, and the magenta frame was used to indicate the responder. Except for the color of the frame, the initiator and responder were presented with the same stimuli.

Six runs, two for fJA, two for sJA and others for control, were conducted in a counterbalanced order across the sessions. The fJA run contained 32 trials, in which the participants switched the role of initiator and responder pseudo-randomly, undertaking each role evenly. The sJA run was identical to the fJA run except for the task. The control run contained 16 trials of the control for fJA and the remaining 16 for sJA. The total number of trials was 192, without jittering of the inter-trial interval.

##### Feature-based JA task (fJA).

**Fig. 2.**
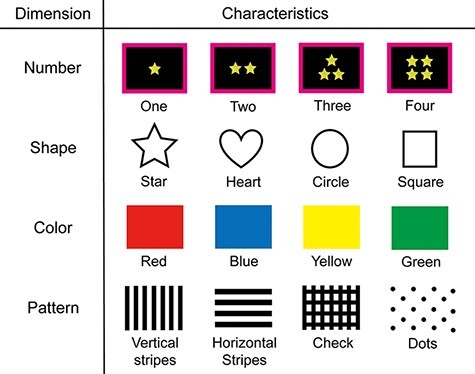
The items of feature-based JA. The objects have four dimensions such as number (1, 2, 3 or 4), shape (star, heart, circle or square), color (red, blue, yellow or green) and pattern (stripe, border, check or dot).

First, a white crosshair representing a gaze fixation point was presented for 2.5 s when ready to start a trial. Next, the target stimulus appeared, and a frame showing the roles of the participants was presented surrounding the target stimulus for 2.5 s. In the fJA task, the 1–4 objects were displayed at the center of the screen. These objects had four dimensions: number (1, 2, 3 or 4), shape (star, heart, circle or square), color (red, blue, yellow or green) and pattern (stripe, border, check or dot; Figure 2). The target stimulus of fJA consisted of the characteristics selected from each dimension, one from each dimension at random and with an equal number of occurrences. The initiator was free to choose one of the four dimensions. Initiators were instructed to choose an unbiased number of dimensions to select before the experiment. After the frame disappeared, the initiator informed the chosen aspect by utterance within 2 s. For example, when the initiator said ‘SHAPE’ during the silent period, the initiator was also required to pay attention to this feature. Then, the responder was required to listen to the initiator’s instruction and said ‘Roger’ (‘Hai’ in Japanese), subsequently attending to the same feature of the dimension of the object in the same silent period. Thereafter, a white gaze point appeared again for 2.5 s and then the gaze point turned red, after which the responder replied stating the characteristic of this dimension within 2 s. The initiator judged whether it was the same object dimension as they saw and fed back verbally (‘correct’ or ‘incorrect’) in the same silent period (Figure 1A and Supplementary Figure S1A).

##### Spatial JA task (sJA).

In each trial, a white crosshair was first presented for 2.5 s. Next, the target stimulus appeared, and a frame showing the roles of the participants was presented surrounding the target stimulus for 2.5 s. In the sJA task, four gray target objects (star, heart, circle or square) were displayed above, below, left and right from the center of the screen. The initiator was free to choose one of the four objects. When the frame disappeared, the initiator uttered or verbally instructed the place of the attended target (i.e. above, below, left or right) while looking at the object for 2 s. The responder was required to listen to the initiator’s instruction and say ‘Roger’, while looking at the same object in the same period. Thereafter, a white gaze point was presented again for 2.5 s, and then the gaze point turned red, after which the responder replied regarding the shape of the object within 2 s. The initiator judged whether the answer was correct or not and fed back verbally in the same silent period (Figure 1B and Supplementary Figure S1B).

##### Control task.

We generated two control tasks that were performed by all participants. Each task corresponded to a feature-based attention task (i.e. fCTRL) or spatial attention (i.e. sCTRL) task (Figure 1C and Supplementary Figure S1C). These two tasks repeatedly alternated four times during the trial run. In the control tasks, the frame color was gray, and the participants performed this task without reference to their partners. The headphones were set to hear their voice instead of the partner’s, and the participants were informed that the partner was conducting the same task but carried out the task in solo mode. During fCTRL, the participants read out the instructions when the frame and instruction disappeared. When the gaze point turned red, they uttered the characteristics of the dimension to themselves. Similarly, during sCTRL, the participants uttered the shape of the object that the instructed direction pointed to.

### Data analysis

#### Image preprocessing.

Image preprocessing procedures and statistical analysis were performed using Statistical Parametric Mapping (SPM)12 revision 6685 (Wellcome Centre for Human Neuroimaging, London, UK) implemented in MATLAB 2015a (Mathworks, Natick, MA, USA). After realignment of the EPI images, a mean EPI image was coregistered with the T1-weighted whole-brain 3D MP-RAGE image, and the parameter was then applied to all EPI images. The MP-RAGE image was normalized to the Montréal Neurological Institute (MNI) T1 image template using a segmentation-normalization method. The normalization parameters were applied to all EPI volumes. The final resolution of the normalized EPI images was 2 mm × 2 mm × 2 mm. The normalized EPI images were thereafter spatially smoothed in three dimensions using an 8 mm full-width at half-maximum Gaussian kernel. In order to reduce computational load, residual images were resliced to 3 mm × 3 mm × 3 mm before being used for inter-brain correlation analysis.

### Statistical analysis

#### First-level analysis.

We adopted a summary statistics approach to depict the neural substrates of task-related brain activity as follows. In the individual analyses, we fitted a general linear model to the fMRI data from each participant. Neural activity was modeled with delta functions convolved with a canonical hemodynamic response function. The design matrix included six regressors of interest (fIJA, fRJA, sIJA, sRJA, fCTRL and sCTRL) that were modeled at the onset of each event, and the duration was 5 s covering the role assignment (for 2.5 s) and cue-response phase (for 2.5 s). What followed with intermission of the fixation period (for 2.5 s) was the verification period (oral response and verify phase for 2.5 s) that was modeled as covariate of no interest (Figure 1).

We used a high-pass filter, which comprised the discrete cosine basis function with a cutoff period of 128 s, to eliminate the artifactual low-frequency trend. No global scaling was performed. Serial temporal autocorrelation of the pooled voxels was estimated with a first-order autoregressive model using the restricted maximum likelihood procedure. The obtained covariance matrix was used to whiten the data (Friston *et al.*, 2002). The estimated parameters were calculated by performing the least-squares estimation on the high-pass filtered whitened data and design matrix. The parameter estimates in the individual analyses consisted of contrast images that were used for the group-level analysis.

#### Second-level analysis.

The resulting contrast images for each condition (fIJA, fRJA, sIJA, sRJA, fCTRL and sCTRL) were used for the group analysis. Predefined contrasts in the second-level analysis are shown in Table 1. The fCTRL and sCTRL were used as the baseline for comparison with brain activity in fIJA, fRJA, sIJA and sRJA conditions (Table 1). We first evaluated the neural substrates of the main effect of JA. Then, we showed the activity gradients of IJA and RJA (denoted as JA) based on the IJA > RJA contrast within the regions of the main effect of JA. Next, we utilized conjunction analysis and identified feature-specific and spatial-specific activation regardless of the role of IJA and RJA. For the conjunction analysis, the statistical maps generated in the second-level analysis were used (the contrasts of sIJA, sRJA, fIJA, fRJA, sIJA > fIJA, sRJA > fRJA, fIJA > sIJA and fRJA > sRJA, Table 1 for details on each contrast). The resulting set of voxel values for each contrast constituted a statistical parametric map of the *t*-statistic (SPM{*t*}). The statistical threshold was set at *P* < 0.05 with a family-wise error (FWE) correction at the cluster level for the entire brain (Friston *et al.*, 1996) with the height threshold of *P* < 0.001 (Flandin and Friston, 2019). For anatomical labeling, we used the Atlas of the Human Brain, 4th edition (Mai *et al.*, 2015).

**Table 1.** Predefined contrasts in the second-level analysis

Contrast		sIJA	sRJA	sCTRL	fIJA	fRJA	fCTRL
1	sIJA	1	0	−1	0	0	0
2	sRJA	0	1	−1	0	0	0
3	fIJA	0	0	0	1	0	−1
4	fRJA	0	0	0	0	1	−1
5	sIJA > fIJA	1	0	−1	−1	0	1
6	sRJA > fRJA	0	1	−1	0	−1	1
7	sIJA < fIJA	−1	0	1	1	0	−1
8	sRJA < fRJA	0	−1	1	0	1	−1
9	IJA > RJA	1	−1	0	1	−1	0
10	Main effect of JA	1	1	−2	1	1	−2
Conjunction							
1 & 2 & 5 & 6	Spatial-specific JA						
3 & 4 & 7 & 8	Feature-specific JA						

#### Inter-brain correlation analysis of brain activity in pairs during the JA task.

In the present study, we conducted two inter-brain correlation analyses: beta-series correlation analysis (Rissman *et al.*, 2004; Koike *et al.*, 2019) and residual time-series correlation analysis (Saito *et al.*, 2010; Tanabe *et al.*, 2012).

In the beta-series inter-brain correlation analysis, we used another univariate generalized linear model (GLM) to define functional connectivity. More specifically, in this GLM, each trial was modeled separately, and each run comprised 32 trials. Moreover, the response and verify phases were modeled as regressors of no interest. Other parameters and settings were the same as above. After the first-level analysis, we obtained 33 beta images per run, including one of no interest. The four runs were reordered in the following order: the first fJA run, the second fJA run, the first sJA run and the second sJA run. This reordering process ensured that the task types were consistent across the participants. The order of the beta images in the run remained the task order, so it was possible to compare the real pairs and pseudo pairs. Therefore, we generated beta-image series that represented the variation in activation associated with the JA task.

Using these data, we examined the beta-series correlation by calculating the correlation coefficient of time-series data of beta value in real pairs and pseudo pairs. This procedure was based on the assumption that the mutual interaction of real pairs causes higher inter-individual correlation of their behavior and neural activity (Koike *et al.*, 2019). In this experiment, all 22 real pairs actually performed the tasks together. Based on these data, we artificially generated 462 pseudo-random pairs that did not complete the JA task together. We evaluated the correlation of the beta value changes between the same coordinates in the beta images of the pairs. Correlation values were transformed to *z*-scores, after which they were compared between real pairs and pseudo pairs using a two-sample *t*-test.

Finally, to assess the pair-specific state-related brain activity distinct from the task-evoked activation, we conducted a residual time-series inter-brain analysis (Saito *et al.*, 2010; Tanabe *et al.*, 2012). In this analysis, we obtained residual time-course data by modeling each event in the time-series data with individual regressors and removing the effect of these task-related activities. Furthermore, to preserve autocorrelative characteristics in the residual time-series data, we turned off the serial correlation function of SPM. Using these data, we examined correlations by calculating the correlation coefficient of time-series data of the residual time course in real and pseudo pairs. Thereafter, we used the two-sample *t*-test to assess whether the residual time-course correlation of the real pairs was greater than that of the pseudo pairs. In this analysis, the measurements were assumed to be independent between levels and the measurements in each level were assumed to have unequal variance. The statistical threshold was set at *P* < 0.05 with an FWE correction at the cluster level for the entire brain (Friston *et al.*, 1996) with the height threshold of *P* < 0.001 (Flandin and Friston, 2019)

## Results

### Inter-brain synchronization of brain activity during the JA task

**Fig. 3.**
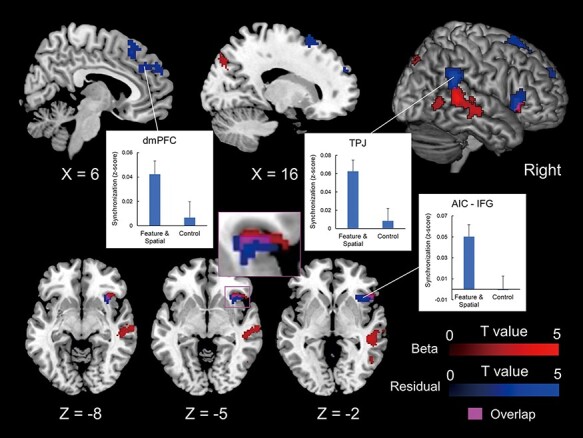
Inter-brain synchronization of the brain activity during the JA task. Task-specific synchronization (beta synchronization, red) and residual synchronization (blue) are superimposed on the template anatomical MRI scan. Their overlap (magenta) is seen in the right AIC. The plot shows standardized correlation value (*z*-score) of the residual time-series synchronization during the JA task condition (feature-based and spatial JA) and control condition in the peak voxels of the dmPFC (x = 6, y = 41, z = 35), TPJ (x = 54, y = −43, z = 29) and the AIC-IFG (x = 48, y = 20, z = 5; S3). Error bars indicate the standard error of the mean.

First, the task-specific beta-series inter-brain correlation was established as the correlation between the IJA-related activation of a participant with the RJA-related activation of homologous regions in the partner (Koike *et al.*, 2019). The right posterior superior temporal sulcus (pSTS), middle temporal gyrus (MTG), IFG-AIC and cuneus were more synchronized in real pairs than in pseudo pairs (red in Figure 3, Supplementary Table S1).

Second, we performed an analysis of the inter-individual neural correlation of residual time-series data after modeling out the task-related activity, representing a synchronization of background activation (Saito *et al.*, 2010; Tanabe *et al.*, 2012). During the JA tasks, inter-individual synchronization in real pairs was significantly higher in the right temporoparietal junction (TPJ), AIC, IFG and mid to caudal part of the dorsomedial prefrontal cortex (dmPFC) than that in pseudo pairs (blue in Figure 3, Supplementary Table S2). There was no inter-individual neural synchronization of residual time-series data during the control condition in which both participants conducted their task without any verbal interaction.

### Task-related activation

**Fig. 4.**
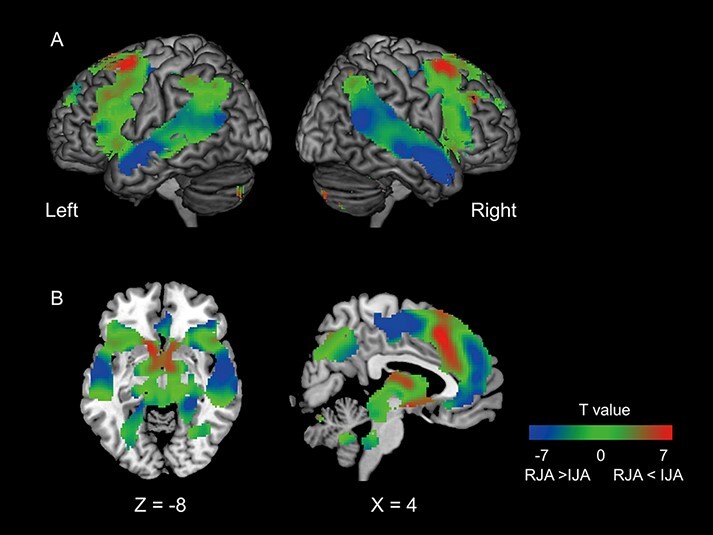
Task-related activation with the IJA- RJA gradation within the main effect of JA regions. IJA-dominant regions are shown in red, RJA-dominant regions in blue and common regions in green.

Figure 4 shows the task-related activation with IJA-RJA gradation based on the IJA > RJA contrast within the main effect of JA regions (Table 1). Common activation by IJA and RJA, irrespective of the targets (spatial- and feature-based), was found in the bilateral AIC to IFG, bilateral MTG and superior temporal gyrus, bilateral precentral gyrus, the supplementary motor area (SMA) to caudal medial superior frontal cortex (mPFC) and anterior cingulate cortex (ACC), left thalamus, left caudate, and midbrain (green).

**Fig. 5.**
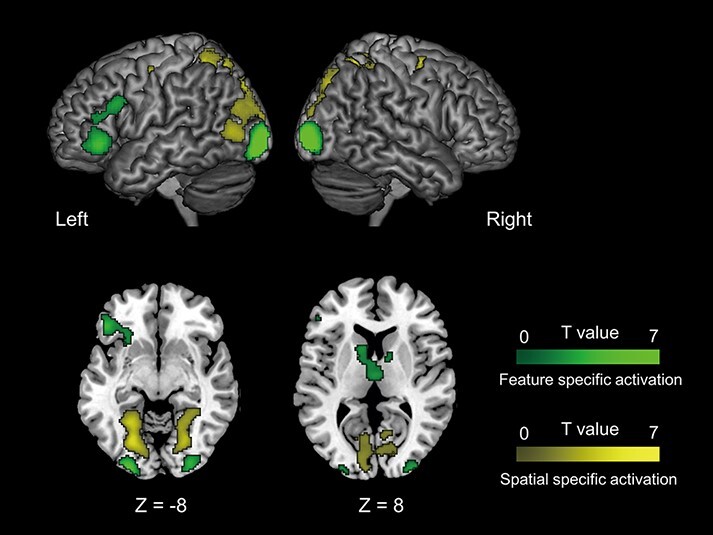
Task-related activation with object specificity. Feature-specific activation (green) and spatial location-specific activation (yellow) are superimposed on the template anatomical MRI scan.

Feature-based specificity, regardless of the role in JA, was found in the left IFG and bilateral inferior occipital poles, observed as brain regions specifically active in fJA rather than sJA (Figure 5 green region, Supplementary Table S3). Similarly, the spatial-location-specific JA task activated the bilateral superior frontal gyrus, lingual gyrus, calcarine gyrus, lateral occipital cortex, cuneus and right postcentral gyrus (Figure 5 yellow region, Supplementary Table S4).

## Discussion

The present study showed pair-specific inter-individual neural synchronization of task-specific activities in the right AIC-IFG, replicating the finding of Koike *et al.* (2019). Furthermore, the right AIC-IFG also showed inter-individual synchronization of the residual time-series data. This synchronization was also found in the right TPJ and the dmPFC comprising the mentalizing network. Considering that the spontaneous neural activity reflects the internal model of the environment (Berkes *et al.*, 2011), the mentalizing network’s synchronization may represent the upper-level forward model of ‘our seeing it’ (monitoring and predicting the goal-directed behavior of self and others), which sends top-down prediction signals to the AIC-IFG where the prediction error generated. Thus, the right AIC-IFG represents the ‘intention in attention’ toward the feature or space, which may send the feedback to the upper-level prediction or internal model of the shared environment represented by the mentalizing network, forming hierarchical representation.

### Synchronization of task-related brain activities

Synchronized fluctuation of task-specific activities was found in the right AIC, extending to the right IFG, and the pSTS; activation of the former region replicated a previous study that used an eye-gaze-mediated JA task (Koike *et al.*, 2019). We identified a single cluster covering both the right AIC and the orbital portion of the IFG. A non-human primate study (Mesulam and Mufson, 1982) showed that the insula is heavily interconnected with the lateral orbital areas and many cortical connections of the lateral orbital cortex are similar to those of the insula. Thus, we referred to this area with synchronized task-related activities as the right AIC-IFG complex. Koike *et al.* (2019) concluded that pair-specific synchronization of task-related activity in the right AIC-IFG complex in humans represents the shared attention. Through internal model mechanisms, spatial attention toward the common reference object can be shared, leading to the identification of each other by aligning their perspectives (Koike *et al.*, 2019). The present findings extend the notion of Koike *et al.* (2019) to verbally mediated JA, in which directedness toward the features of an object are shared even without gaze exchange.

Another task-specific synchronization was found in the pSTS, which is critical in parsing multimodal input sequences into discrete units to extract meaning, commonly observed in both linguistic processing and social perception (Redcay, 2008). Thus, task-specific synchronization of the pSTS is related to sharing semantic knowledge anchored with the visual experience, retrieved in the anterior temporal lobe (Olson *et al.*, 2013). Taken together, the task-specific synchronization in the right pSTS represents shared semantic knowledge of the target (proposition), and this synchronization in the right AIC-IFG complex represents shared epistemic mental states (attitude) toward the target.

### Synchronization of state-related brain activities

We identified residual time-series synchronization in the right AIC-IFG complex, right TPJ and dmPFC. Saito *et al.* (2010) demonstrated residual time-series synchronization of the right AIC-IFG complex with an eye-gaze-mediated JA task. They argued that, by eliminating the task-related activity, the residual time-series data represented the baseline condition, analogous to the resting-state activities (Fair *et al.*, 2007). As Saito *et al.* (2010) utilized eye-gaze-mediated RJA, their baseline condition was real-time eye contact, and the right AIC-IFG synchronization represents sharing the intentionality of ‘I am attending to you’. In this study, real-time eye contact was eliminated while the belief, ‘I see it as part of our seeing it’, was shared, which was anchored by the attention toward the object the participants were looking at. Thus, the baseline state in this study was sharing the belief of sharing the situation. This belief may be regarded as ‘allyship,’ according to the recent work by Lau *et al.* (2020). They showed that the right AIC-IFG complex and the DMN, including the retrogenual ACC, are involved in the process of distinguishing ‘us’ by tracking interpersonal similarities. The dmPFC, TPJ, lateral temporal cortex and temporal pole constitute the subsystem of the DMN, which play a role in introspecting about the mental states of both self and others (Andrews-Hanna, 2012). Thus, the present finding of inter-individual synchronization of the right AIC-IFG complex and the DMN may form the neural basis of the first-person plural perspective (‘we-mode’) (Gallotti and Frith, 2013). This notion is supported by the finding that no residual synchronization was observed across the whole brain during the control condition when no verbal JA task was conducted (Figure 3).

Previous hyperscanning fMRI with JA task by Bilek *et al.* (2015) found the inter-individual synchronization in the right TPJ. The anterior cluster of the right TPJ (rTPJa) is connected to the salience network, i.e. the AIC and ACC, and the posterior cluster (rTPJp) is connected to the DMN, i.e. the mPFC and posterior cingulate cortex (Kubit and Jack, 2013). Anterior–posterior functional differentiation has also been reported, supporting the notion that the rTPJa is associated with attention control domains and the rTPJp with those of beliefs (Decety and Lamm, 2007; Mitchell, 2008; Mars *et al.*, 2012; Bzdok *et al.*, 2013; Krall *et al.*, 2015). Informational flow from the rTPJa to the rTPJp was found during cooperation (Abe *et al.*, 2019). These studies suggest that the collaboration between the rTPJp and rTPJa is critical in linking self and other-related information (Bzdok *et al.*, 2013). This study provides additional evidence that the information derived from shared experiences originates from the anterior portion of the STS and is transmitted rostrocaudally to reach the right TPJ region.

The mPFC has a functional gradient along the ventro-dorsal axis from self to others (Mitchell *et al.*, 2006; Denny *et al.*, 2012). According to Denny *et al.* (2012), our synchronized residual time series is in the other-related judgment region. The dmPFC plays a role in adopting another person’s perspective and comparing self-other perspectives (Ruby *et al.*, 2007), in conjunction with the TPJ and the posterior cingulate cortex (Denny *et al.*, 2012). Furthermore, the anterior region, located in Brodmann area 9, is related to self-other unity during cooperative and competitive tasks (Wittmann *et al.*, 2016). Appreciation of self and other perspectives is critical for sharing information (Mundy, 2018). Baek *et al.* (2017) observed ventromedial PFC activation when participants decided a headline was interesting to themselves (self-referenced processing), but a distributed system of dmPFC and TPJ activation was observed when participants decided to share a headline (self- and other-referenced processing). Van Overwalle and Baetens (2009) suggested that the mPFC is a module that integrates social information across time at an abstract cognitive level. The dmPFC is related to the processing of socially or emotionally relevant information (Saxe and Powell, 2006) to infer enduring propositions of others and self. Thus, enhanced neural synchronization of the residual time series of the dmPFC in conjunction with the right TPJ and AIC-IFG complex in this study represents the shared contextual information relevant to infer the partner’s utterance, that is, the belief of ‘I am seeing it as part of our seeing it’, leading to the sharing of visual experiences.

### Implications for the development of social capability

JA is postulated to be the precursor of the theory of mind, aka, mentalizing (Baron-Cohen, 1995). JA emerges around the age of 1 year, whereas mentalizing emerges at around the age of 4 years when children pass the false-belief task. As the triadic representation relationship involves shared attitude (attend, goals, desire, etc.), Baron-Cohen (1995) hypothesized that mentalizing is triggered in development by taking triadic representations from JA and converting them into M-representations through linguistic interaction. A longitudinal study showed that gaze following a critical component of JA, at 10.5 months of age, predicted the use of mental-state terms at 2.5 years; the latter predicted the theory of mind at 4.5 years (Brooks and Meltzoff, 2015). The authors hypothesized that gaze following fueled children’s linguistic coding of psychological concepts, which in turn supported the ontogenesis of an explicit ‘theory of mind’. JA requires participants to monitor and represent both their own goal-related intentional activity and of others (Mundy and Newell, 2007). This study showed that the forward models of intention and directedness of attention are hierarchically represented, in which hierarchy the right AIC is the node to link them. This finding supports a long-standing theory about the relations between JA and social-cognitive development toward mentalizing (Baron-Cohen, 1989; Mundy, 1995, 2003; Tomasello, 1995; Tomasello *et al.*, 2005; Mundy and Newell, 2007).

### Specific regions activated by object feature and spatial tasks

In this study, we conducted an orally mediated JA task that eliminated eye-gaze processing. As eye-gaze provides rich social information, it is crucial to segregate eye-gaze-related activation from the sharing of the attention per se. Two type categories were shared: space and feature. The space category effect found in the parieto-prefrontal network is consistent with its function in top-down spatial attention (Corbetta *et al.*, 2008). The superior frontal gyrus (Brodmann area 8, frontal eye field (Beauchamp *et al.*, 2001)) and lateral occipital cortex (Corbetta *et al.*, 1998) have been regarded as brain regions relevant to spatial attention. The feature category effect was found in the orbital part of the left IFG, consistent with its function in semantic categorization (Kapur *et al.*, 1994; Gabrieli *et al.*, 1996; Wagner *et al.*, 1997; Dapretto and Bookheimer, 1999). The orbital part of left IFG, where activity was seen in our study, is involved in controlling access to lexical items in semantic memory (Badre and Wagner, 2007) and in retrieving vocabulary items with related features (Ye and Zhou, 2009). These findings suggest that the categories of an attentional target (feature and space) are represented separately.

### Specific regions activated in each of the IJA and RJA roles

IJA-specific activation was found in the caudal mPFC to the ACC and the ventral striatum, consistent with previous studies (Schilbach *et al.*, 2010; Redcay *et al.*, 2012; Caruana *et al.*, 2015; Koike *et al.*, 2019). Koike *et al.* (2019) showed that the ACC is involved in the volitional selection of a target object during IJA. The authors argued that together with the AIC as a saliency network, the ACC is likely involved in the top-down direction of one’s gaze and attention when the target is selected during IJA. Schilbach *et al.* (2010) reported that the ventral striatum was activated during IJA and concluded that control over the other person by initiating JA is intrinsically rewarding, thus JA may affect the social interaction. Unfortunately, we did not measure the behavioral change of the participants before and after the experiments. Future study is warranted for exploring JA’s effect on the quality of the social relationship. The present and previous findings support the notion that the major component of IJA is an awareness of attention to self (Bates *et al.*, 1975; Reddy, 2003; Kim and Mundy, 2012; Edwards *et al.*, 2015) and that expression of positive affect is often accompanied by IJA, but not with other forms of JA behaviors (Kasari *et al.*, 1990; Mundy *et al.*, 1992). Thus, the present findings are concordant with the motivation theory of JA (Mundy, 1995, 2018; Tomasello *et al.*, 2005), postulating that IJA involves self-referenced processing of the predicted social reward value of attention shifts of the partner (Mundy, 2003; Mundy *et al.*, 2009).

The RJA-specific activation pattern overlaps with that of the mentalizing network, comprising the anterior and posterior STS, ventromedial PFC, TPJ and SMA/pre-SMA. The RJA task requires the responder to attend to the feature category (for example, color) or location of the object specified by the initiator. Correctly specifying the feature of the object or its location requires reasoning about the initiator’s intention, that is, mentalizing; this consequently leads to the retrieval and utterance of the name from the semantic knowledge or concept. A recent meta-analysis of the mentalizing literature (Schurz *et al.*, 2014) indicated that the mPFC and bilateral TPJ are the core that is activated whenever reasoning about mental states is evoked. The core activation is surrounded by task-specific activation in the anterior temporal lobe, critical for storing and retrieving semantic knowledge (Olson *et al.*, 2013). The STS is critical in extracting the meaning by integrating the discrete units, which are derived from parsing the sequences of multimodal input (such as voice or motion), commonly observed in both linguistic processing and social perception (Redcay, 2008). Thus, the posterior and anterior sectors of the STS are likely related to the integration of visual experience with semantic knowledge.

## Limitation and future perspective

Redcay and Schilbach (2019) reviewed the methods for elucidating social cognition’s interactive nature, pointing out that the hyperscanning fMRI has its edge in depicting inter-individual synchronization as an emergent phenomenon that cannot be reduced to individual, such as ‘sharing’. In the present study, we have shown that the sharing of visual experience is represented by the sharing of the hierarchically organized prediction, or forward model, whose neural underpinning was represented as inter-individual synchronization. Hyperscanning experiments with electroencephalogram or near-infrared spectroscopy in more ecological situation is warranted for future study.

## Conclusion

Verbally shared visual experience is represented by the moment-to-moment synchronization of the AIC–pSTS, with the shared context represented by the state-related residual synchronization of the DMN. The present findings indicate that the shared visual experience is represented by neural synchronization of the DMN, hierarchically linked with the right AIC as the core representation of the JA within the limbic mirror system and salience network.

## Supplementary Material

nsab082_Supp

## Footnotes

1
Searle (1983, pp. 3) utilized the capitalized word ‘Intentionality’ in order to clarify the difference between intentionality and intention: ‘Intentionality is directedness; intending to do something is just one kind of Intentionality among others’.
