# Supplementary material for: Neural substrates of shared visual experiences: a hyperscanning fMRI study
Source: Soc Cogn Affect Neurosci. 2021 Jun 28;16(12):1264–75. doi: 10.1093/scan/nsab082 (PMC8717063; doi:10.1093/scan/nsab082)
Supplement: nsab082_Supp [file nsab082_supp.zip › scan-21-010-File008.docx]

**
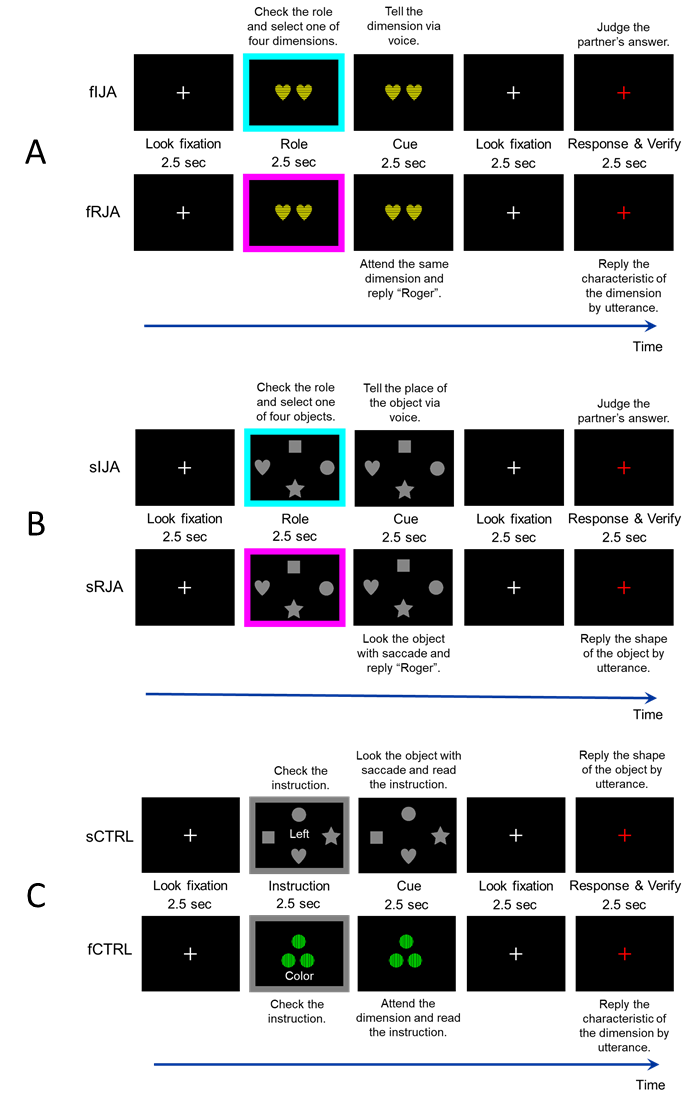
**

**Figure S1.** Illustration of the time course in a trial of task conditions. (A) feature-based joint attention task, (B) spatial joint attention task, (C) feature-based and spatial attention control (solo) task.

**Table S1.** The significant clusters of inter-brain beta series correlation analysis [real pair > pseudo-pair] (the volume of one brain activity cluster, MNI coordinates (x, y, z), cluster level p-values (FWE corrected), maximum T-value, hemisphere, and the locations of local maxima were defined based on Mai et al. (2015)

| Volume  (mm^3^) | MNI coordinates | | | *p*-value  (FWE-corr) | T-value | hemisphere | Location |
| --- | --- | --- | --- | --- | --- | --- | --- |
|  | x | y | z |  |  |  |  |
| 4584 | 52 | -38 | 6 | <0.001 | 5.47 | right | middle temporal gyrus |
|  | 58 | -24 | 4 |  | 3.51 | right | superior temporal gyrus |
| 744 | 46 | -56 | 4 | 0.016 | 4.28 | right | middle temporal gyrus |
|  | 50 | -60 | -4 |  | 3.53 | right | inferior temporal gyrus |
|  | 38 | -58 | 4 |  | 3.35 | right | inferior longitudinal fascicle |
| 680 | 34 | 24 | -8 | 0.024 | 3.96 | right | basal operculum |
|  | 48 | 22 | -4 |  | 3.87 | right | inferior frontal gyrus, trianglar part |
| 992 | 16 | -78 | 40 | 0.003 | 3.93 | right | precuneus |
|  | 18 | -80 | 48 |  | 3.22 | right | superior parietal lobule |

FWE, family-wise error rate; MNI, Montréal Neurological Institute.

**Table S2.** The significant clusters of inter-brain residual time series correlation analysis [real pair > pseudo-pair].

| Volume  (mm^3^) | MNI coordinates | | | *p*-value  (FWE-corr) | T-value | hemisphere | Location |
| --- | --- | --- | --- | --- | --- | --- | --- |
|  | x | y | z |  |  |  |  |
| 3699 | 54 | -43 | 29 | <0.001 | 5.16 | right | post. transverse temporal gyrus |
| 2781 | 48 | 20 | 5 | <0.001 | 4.39 | right | frontal operculum |
|  | 39 | 20 | -1 |  | 4.29 | right | insular pole |
|  | 30 | 14 | -13 |  | 3.82 | right | area piriformis insulae |
| 2673 | 9 | 26 | 59 | <0.001 | 4.30 | right | superior frontal gyrus, lateral part |
|  | 6 | 26 | 50 |  | 4.27 | right | superior frontal gyrus, medial part |
| 1485 | 6 | 41 | 35 | 0.008 | 3.81 | right | superior frontal gyrus, medial part |
|  | 9 | 59 | 29 |  | 3.69 | right | superior frontal gyrus |

FWE, family-wise error rate; MNI, Montréal Neurological Institute.

**Table S3.** The significance cluster of the specific active region in the feature-based joint attention condition.

| Volume  (mm^3^) | MNI coordinates | | | *p*-value  (FWE-corr) | T-value | hemisphere | Location |
| --- | --- | --- | --- | --- | --- | --- | --- |
|  | x | y | z |  |  |  |  |
| 2848 | -26 | -94 | -8 | 0.007 | 6.86 | left | inferior lingual gyrus, lateral part |
| 3344 | 28 | -94 | -4 | 0.003 | 6.36 | right | fusiform gyrus, post. Part |
|  | 30 | -96 | 4 |  | 6.09 | right | inferior occipital gyrus |
| 4584 | -50 | 32 | -4 | <0.001 | 5.90 | left | inferior frontal gyrus, orbital part |
|  | -26 | 20 | -12 |  | 4.38 | left | posterior orbital gyrus |
| 3168 | -50 | 18 | 26 | 0.004 | 4.81 | left | inferior frontal gyrus, opercular part |
|  | -48 | 34 | 16 |  | 3.89 | left | middle frontal gyrus |
| 2672 | 2 | -12 | 8 | 0.010 | 4.27 | right | internal medullary lamina of the thalamus |
|  | -10 | 10 | 8 |  | 4.24 | left | medial caudate |
|  | -4 | -4 | 6 |  | 3.75 | left | stria medullaris of thalamus |
|  | 14 | 2 | 10 |  | 3.69 | right | anterior limb of the internal capsule |
|  | 12 | 0 | -4 |  | 3.56 | right | ventral globus palidus |
|  | 8 | 0 | 4 |  | 3.53 | right | bed n. of the stria terminalis, central div. |

FWE, family-wise error rate; MNI, Montréal Neurological Institute.

**Table S4.** The significance cluster of the specific active region in the spatial joint attention condition.

| Volume  (mm^3^) | MNI coordinates | | | *p*-value  (FWE-corr) | T-value | hemisphere | Location |
| --- | --- | --- | --- | --- | --- | --- | --- |
|  | x | y | z |  |  |  |  |
| 4904 | 26 | -6 | 50 | <0.001 | 7.17 | right | superior frontal gyrus, lateral part |
| 41336 | -22 | -70 | -4 | <0.001 | 7.12 | left | fusiform gyrus |
|  | 20 | -68 | -4 |  | 6.47 | right | fusiform gyrus |
|  | -20 | -80 | 40 |  | 5.75 | left | precuneus |
|  | -14 | -80 | 0 |  | 5.40 | left | inferior lingual gyrus, lateral part |
|  | 20 | -76 | 42 |  | 5.08 | right | Arcus parieto-occipitalis, ant. division |
|  | 14 | -56 | 54 |  | 4.92 | right | precuneus |
|  | -16 | -86 | 22 |  | 4.90 | left | occipital gyrus |
|  | -20 | -66 | 16 |  | 4.68 | left | striate area |
|  | -24 | -82 | 22 |  | 4.64 | left | superior longitudinal fascicle I, dorsal comp. |
|  | -10 | -96 | 20 |  | 4.61 | left | superior occipital gyrus, medial part |
|  | 18 | -90 | 22 |  | 4.50 | right | Arcus parieto-occipitalis, post. division |
|  | 34 | -36 | 46 |  | 4.06 | right | postcentral gyrus |
|  | 26 | -50 | 60 |  | 4.04 | right | superior parietal lobule |
|  | 22 | -64 | 22 |  | 4.00 | right | occipital gyri |
|  | -8 | -94 | 12 |  | 3.77 | left | middle sagittal gyrus |
|  | -36 | -76 | 24 |  | 3.66 | left | superior longitudinal fascicle II, central comp. |
|  | 40 | -26 | 38 |  | 3.50 | right | superior longitudinal fascicle III, vent. comp. |
|  | -38 | -84 | 24 |  | 3.32 | left | medial occipital gyrus |
| 3592 | -22 | -6 | 52 | 0.002 | 6.99 | left | superior frontal gyrus, lateral part |
| 3168 | -22 | -50 | 60 | 0.004 | 4.87 | left | superior parietal lobule |
|  | -12 | -54 | 62 |  | 3.79 | left | precuneus |

FWE, family-wise error rate; MNI, Montréal Neurological Institute.
